# Manipulation of gold colloidal nanoparticles with atomic force microscopy in dynamic mode: influence of particle–substrate chemistry and morphology, and of operating conditions

**DOI:** 10.3762/bjnano.2.10

**Published:** 2011-02-04

**Authors:** Samer Darwich, Karine Mougin, Akshata Rao, Enrico Gnecco, Shrisudersan Jayaraman, Hamidou Haidara

**Affiliations:** 1 1IS2M-CNRS, 15 Rue Jean Starcky, 68057 Mulhouse, France; 2 Institute of Physics, University of Basel, and NCCR “Nanoscale Science”, Klingelbergstrasse 82, 4056 Basel, Switzerland; 3 Corning Incorporated, 1 Science Center Road, Corning, NY 14831, USA

**Keywords:** atomic force microscopy, intermolecular interaction, manipulation, nanoparticles, precise positioning, self-assembled monolayers

## Abstract

One key component in the assembly of nanoparticles is their precise positioning to enable the creation of new complex nano-objects. Controlling the nanoscale interactions is crucial for the prediction and understanding of the behaviour of nanoparticles (NPs) during their assembly. In the present work, we have manipulated bare and functionalized gold nanoparticles on flat and patterned silicon and silicon coated substrates with dynamic atomic force microscopy (AFM). Under ambient conditions, the particles adhere to silicon until a critical drive amplitude is reached by oscillations of the probing tip. Beyond that threshold, the particles start to follow different directions, depending on their geometry, size and adhesion to the substrate. Higher and respectively, lower mobility was observed when the gold particles were coated with methyl (–CH_3_) and hydroxyl (–OH) terminated thiol groups. This major result suggests that the adhesion of the particles to the substrate is strongly reduced by the presence of hydrophobic interfaces. The influence of critical parameters on the manipulation was investigated and discussed viz. the shape, size and grafting of the NPs, as well as the surface chemistry and the patterning of the substrate, and finally the operating conditions (temperature, humidity and scan velocity). Whereas the operating conditions and substrate structure are shown to have a strong effect on the mobility of the particles, we did not find any differences when manipulating ordered vs random distributed particles.

## Introduction

Nanotechnology, which aims at the ideal miniaturization of devices and machines down to atomic and molecular sizes has become a strategic topic with a promising future in high technology for the forthcoming century [1]. By the precise control of atoms, molecules, or nanoscale objects, new sensors and man-made materials, micromachines, organic integrated computers, microscale intelligence system, communication tools would be possible within the near future [2]. However, for new nanotechnology products, there are still many problems to be solved such as nanomanipulation which has a great impact on nanometer scale expertise. By manipulation of nanoscale objects (nano-objects), we mean using external force for positioning or assembling objects in two (2-D) or three (3-D) dimensions by twisting, bending, picking-and-placing, or pushing and pulling them [3]. Nanomanipulation is a complex 3-D problem. Because mechanical and chemical properties of substrates, probing tools and nano-objects (especially ‘particles’) are combined, different results are expected depending on the environmental and operating conditions. Numerous methods exist for the manipulation of nanostructures and can be classified into two categories as non-contact and contact manipulation systems. In the former, laser trapping (optical tweezers) or electrostatic or magnetic field forces are utilized. Thus, Yamomoto et al. [4] cut DNA using restriction enzymes on a laser trapped bead, Vonna et al. used magnetic tweezers and beads to stretch cell membranes [5] and Stroscio et al. [6] utilized electrical forces between a scanning tunneling microscopy (STM) probe tip and surface atoms for manipulating Xe or Ni atoms. More precisely, manipulation of nanoparticles (nanoscale metal particles (NPs)) in a non-contact mode was the first approach to manipulate these nano-objects. Historically, the first accurate manipulation studies of nanoparticles were performed by STM. In a pioneer experiment, Cuberes et al. moved single C_60_ molecules along the steps of a Cu(111) surface using an STM in UHV [7]. In addition, the majority of the STM experiments were performed at cryogenic temperatures [8]. Unfortunately, despite the accurate level of control obtained with STM, the energy dissipated in the manipulation process cannot be estimated by this technique. Recently, it has been shown that another scanning probe technique, atomic force microscopy (AFM), is capable of positioning single atoms or clusters even at room temperature, and has thus become popular as a simple manipulation tool [9–10]. Changing its function from only imaging to both imaging and manipulation, new challenging problems did arise. Three main modes are used in atomic force microscopy, i.e., non-contact (NC) mode, contact mode and intermittent tapping mode.

The first mode used in AFM was the contact mode. Manipulation of large C_60_ islands on NaCl was performed by Lüthi et al. using contact AFM [11]. Even if the shear between islands and crystal surface can be derived from the frictional forces experienced by the AFM tip while scanning, the applicability of contact AFM to nanomanipulation was limited to relatively large objects (tens of nanometers in size). The latest results obtained by Custance et al. show that it is now possible to manipulate single atoms using NC-AFM [12]. Byungsoo Kim et al. [13] have also proposed a new explanation for the extraction and deposition of atoms using AFM. In the contact mode, different strategies, such as pushing and pulling, have been used to manipulate nanoclusters. Firstly, the tip can be used for positioning particles on a substrate by pushing or pulling operations [14–15]. For instance, M.C. Strus et al. have manipulated carbon nanotubes and estimated the flexural strain energy distributions and static frictional force between a carbon nanotube and a SiO_2_ surface [16]. Nanometer scale antimony particles have been manipulated on an atomically flat graphite surface by atomic force microscopy techniques and quantitative information on interfacial friction was extracted from the lateral manipulation of these nanoparticles [17]. These particles were first *pushed* on a graphite surface by the AFM tips and then manipulated by placing the AFM tip *on top* of the particles. Above a certain lateral force threshold, particle sliding was observed, which has allowed the transition from static to kinetic friction to be quantified [18].

A compromise between the contact and non-contact AFM techniques is the intermittent mode, the so called tapping mode. In this mode the phase shift of the cantilever oscillations with respect to the external periodic excitation can be used to estimate the dissipated energy during manipulation. This method was recently used by Ritter and coworkers to manipulate antimony particles on a graphite surface in air [17–18]. Paollicelli et al. manipulated gold nanoparticles deposited on highly oriented pyrolitic graphite using AFM in tapping mode. NPs were selectively moved as a function of their size varying from 24 up to 42 nm in diameter and the energy detachment threshold of NPs was estimated accordingly [19]. Sitti and coworkers have also manipulated nanoscale latex particles positioned on Si substrates with an accuracy of about 30 nm [20] whilst Mougin et al. manipulated as-synthesized and functionalized gold nanoparticles on silicon substrates with dynamic AFM [21]. In all these techniques, the major difficulties that arise are related to the quantification of the dynamical processes occurring during manipulation, i.e., collisions between probing tips and particles, friction between particles and substrates, electrostatic interactions among all of them, etc.

For this reason, colloidal particles have appeared as model nano-objects because they can be produced in various well-controlled sizes and from various materials such as metals or semiconductors. Of particular interest has been the use of metal nanoparticles, which have been applied to the development of highly sensitive nanoparticle-based detection assays that utilize electrical or optical detection (colorimetric and surface enhanced Raman spectroscopy (SERS)). For different reasons gold particles are particularly attractive in this field. For instance, they are ideal electrodes for molecular electronics [22]. Gold clusters below 5 nm in size deposited onto thin metal oxides also exhibit unexpected highly catalytic activity (not obtained with bulk metal) for different types of reactions, e.g., combustion, hydrogenation, reduction etc. [23–24]. Coated with organic molecules, gold nanoparticles can be used for DNA assays in genomics [25–26], as signal amplifiers for biological recognition or as quantitation of tags in biological assays. To utilize and optimize the chemical and physical properties of gold NPs, a great deal of research has been done regarding the control of size [27–28], shape [29–30], surface chemistry [31–32] and aggregation morphology of nanoparticles as isolated clusters [33], or as single- or multilayer coatings [34]. The manipulation of nanoparticles, especially colloidal gold NPs, by AFM can be inﬂuenced by the structural characteristics of the particle, tip and surface, in particular the intermolecular interactions between tip and particle or particle and surface. In addition, both the physical structure of the substrate (topography) and the operating conditions (environmental conditions and scan velocity of the tip [35]) determine to a large extent the tip–particle–substrate interactions and behavior. Furthermore, the fundamental understanding of the different types of particle motion during manipulation, such as sliding, rolling, stick-slip and spinning, is crucial since the mode of motion of particles determines the energy loss and wear in the contacting surfaces.

In this paper, the sensitivity of those critical parameters on the mobility of gold nanoparticles during their manipulation using AFM in tapping mode has been investigated. In particular, the effects of the size, shape and coating of the nanoparticles, the lateral scan velocity, the particle-surface interactions and the environmental conditions, especially temperature *T* and relative humidity *RH*%, are presented and discussed. The dependency of the energy dissipation during the manipulation was particularly studied as a function of size, coating of particles, substrate and temperature. Finally, interpretation of the physico-chemical mechanisms involved at both interfaces – tip–particle and particle–surface – during the movement of the particle was proposed and partially verified by modeling; nevertheless additional investigations are still needed.

## Results and Discussion

Spherical and asymmetrical gold nanoparticles were synthesized as described in the Experimental section and deposited onto flat and patterned surfaces. Accurate manipulation was performed using AFM in tapping mode as it provides indirect access to dissipation energy during particle movement [21]. Since the same microscope is used to either image or manipulate at a given instant, imaging is almost impossible while pushing the nanoparticle. To face this problem, imaging is carried out before and after manipulation using a fixed reference to locate the final position of the particle.

The ﬁrst part of the discussion will focus on the influence of the size and shape of the particle on manipulation. Then, we will examine the effect of functional (hydrophilic vs hydrophobic) molecules grafted on the Au nanoparticles on their mobility. In addition, we will address the important issue of environmental conditions (*T*, *RH*%), surface topography and tip scan velocities on the manipulation performance of gold nanoparticles. Finally, conclusions with discussions and future directions are given in the last paragraph.

### 1. Influence of size and shape of the particle

#### A. Influence of the size of the spherical Au particle

Sizes of gold spherical nanoparticles(NPs) were tuned from 5 nm up to 65 nm according to the synthesis procedure described in the Experimental section. “As-synthesized” Au NPs, meaning NPs covered with citrate stabilizing group (COO^−^), referred to as “reference NPs” were deposited onto bare and hydrophobized (CH_3_-terminated coating) silicon wafers, and manipulated using AFM in tapping mode. During manipulation, the oscillation amplitude of the tip, *A*_set_, was kept constant by a feedback loop. In such cases, the power dissipation accompanying the tip-sample interaction can be determined from the following relationship [21–36]:

[1]
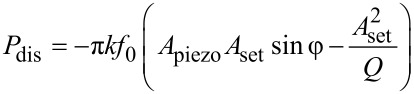


where *A*_piezo_ is the oscillation amplitude of a piezo-element coupled to the cantilever, *f*_0_, *k* and *Q* are the resonance frequency, the spring constant and the quality factor of the free cantilever, respectively, and 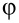 is the phase shift caused by the interaction between the tip and the underlying particles or surface.

**Figure 1:**
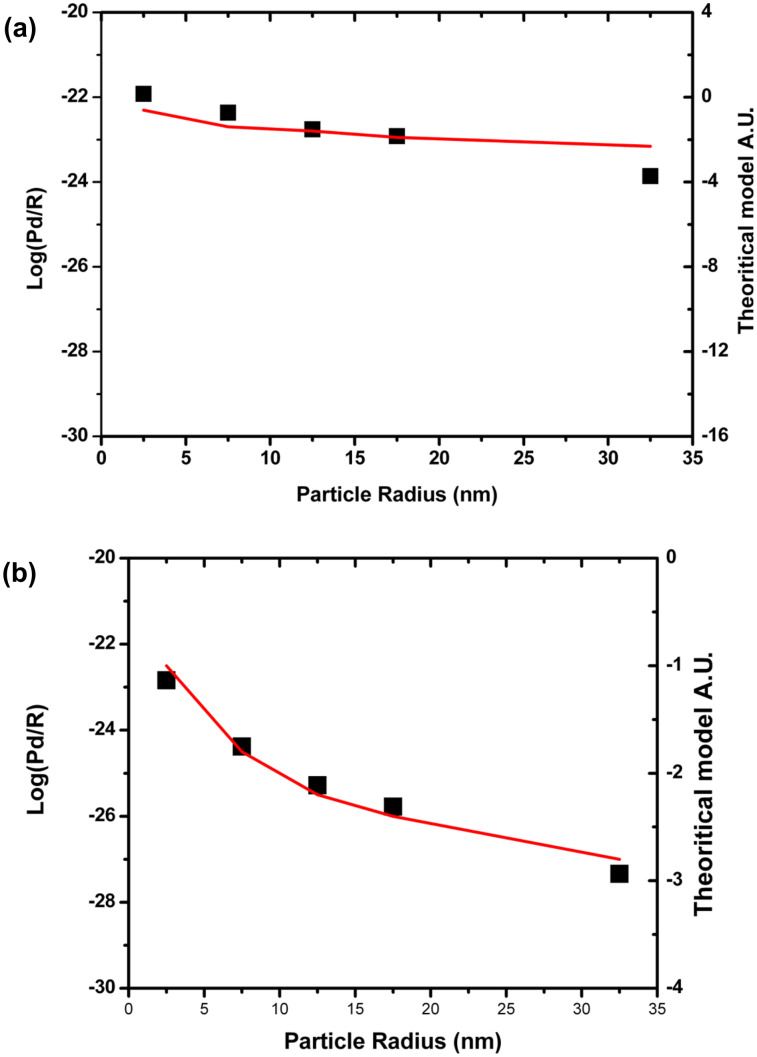
Evolution of the logarithm of the dissipated power normalized by the radius (*R*) as a function of (a) as-synthesized spherical Au nanoparticles on bare silicon wafer versus the particle radius *R* (squares: experimental data; solid line: theoretical data) corresponding to a pure sliding model and (b) spherical Au nanoparticles on silicon wafer coated with –CH_3_ terminated groups (hydrophobic coating) versus the particle radius *R* (squares: experimental data, solid line: theoretical data) corresponding to a pure rotation model. Both after the tap of a tip in a typical AFM tapping mode manipulation as described by Sitti [37–38].

**Scheme 1:**
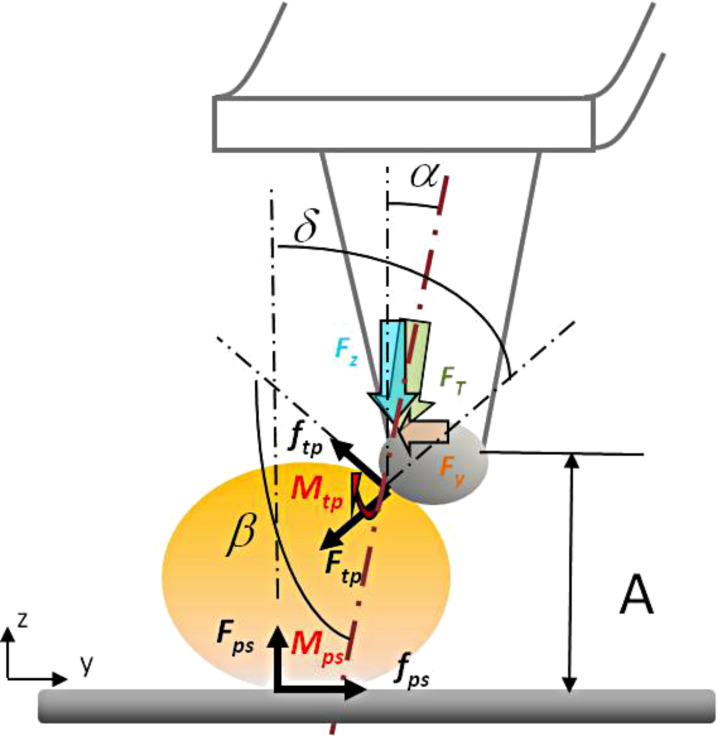
Scheme presenting the different forces during tip–particle and particle–substrate interactions, and the angles α, β and δ.

The calculation of the dissipated power (*P*_dis_) was performed for 5 sizes of Au colloidal particles whose radius (*R*) was varied from 5 up to 65 nm. Figure 1a and Figure 1b show a logarithmic plot of the dissipated power normalized by the radius of the nanoparticle as a function of the particle radius, on bare and hydrophobic coated silicon wafers, respectively. These plots actually can be fitted using an approximation of a friction model for NPs rolling and sliding on the substrate [37–38]. The red curves describe simulated dynamic behavior of the nanoparticles according to pure sliding (Figure 1a) and rotation (Figure 1b) models of the nanoparticle in a typical AFM tapping mode manipulation as described by Sitti [37–38]. According to this model, the force brought by the tip to the particle should be higher than a threshold value given by


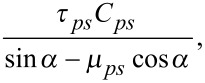


for sliding and


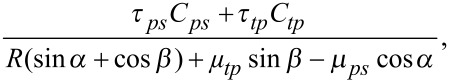


for rolling of the NP. In these expressions, μ is the friction coefficient*,* τ is the shear strength, *C* the contact area, and α and β are the angles which are defined in Scheme 1. The subscripts tp and ps as of τ , *C* and μ represent the tip–particle and particle–substrate contacts, respectively.

These results thus display the dependence of the movement of the particle on, both, their size and the substrate–surface chemistry, underscoring in particular the importance of the particle–substrate interactions on the mobility and behavior of nano-objects on manipulation.

**Figure 2:**
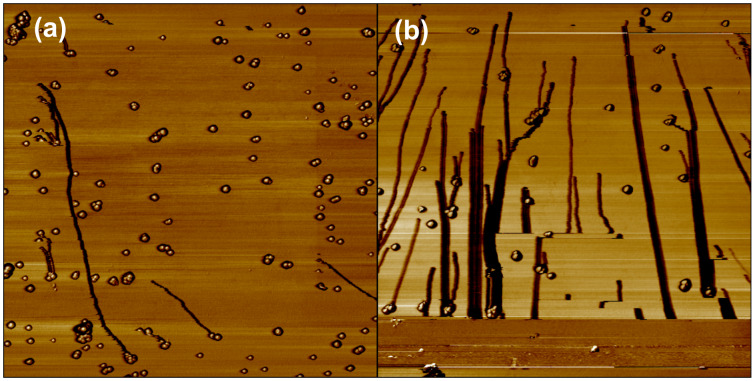
Typical trajectories of bare gold nanoparticles (20 nm diameter) on a silicon substrate when the probing tip moves along a zigzag path: (a) low drive amplitude, (b) high drive amplitude. Scan size: 5 µm.

**Figure 3:**
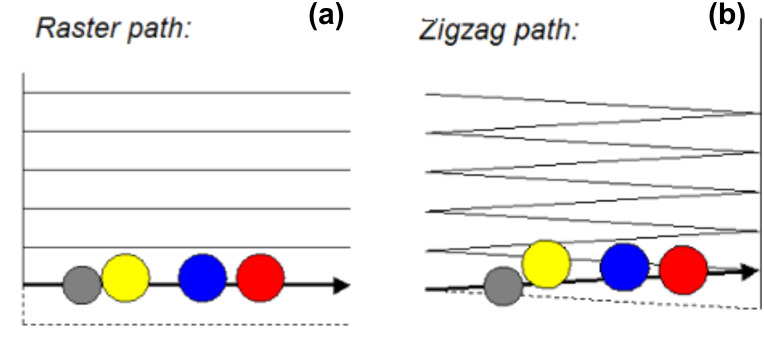
Typical scan patterns used in AFM: (a) raster scan path used by Nanosurf (b) zigzag scan path used by Veeco. *Top view:* the grey disk corresponds to the position of the tip on the surface and the yellow, blue and red disks are the positions of spherical particles pushed by the tip along its scan path.

Although crucial, these particles–substrate interactions actually represent one parameter among other important physical parameters. Indeed small and large particles do not undergo the same trajectory during manipulation. This size-dependence of the particle trajectory under manipulation can thus provide a way to fractionate or to separate a mixture of nano-objects. In Figure 2a and Figure 2b, we can observe that large (a few dozens of nanometers) particles move at a small angle with respect to the normal of the tip's fast scan direction, until they reach the bottom of the scan area, whereas smaller ones slide to the edge of the scan area using a shorter path. From this observation it is possible to fractionate and separate small from big particles adsorbed on a substrate. This size-dependence of the particle trajectory was explained by a simulation which shows that the trajectory of the particle at the same time depends on i) the operating parameter which is the scanning path used by AFM (zigzag or scattered one, Figure 3), ii) the density of scan lines and, iii) the parameter *R*_tot_ which corresponds to the sum of the radii of the tip and the particle [39].

Indeed, it has been observed (Figure 2b) that two particles that collide at a point and move together can be considered as a single particle. If we compare two consecutive trajectories of the particle before and after collision, the single Au particle (thinner line) moves at a smaller angle, as compared to the case where it meets another particle (thicker line). In this case, the variation of the trajectory can be explained by the variation of the radius of the average cluster *R*_tot_ (different sizes move at different angles).

Moreover, the modeling of the NPs trajectory addresses a relation between the frictional forces acting on spherical nanoparticles, and the trajectories predicted. This model can also be used to interpret the trajectory fluctuations and the apparent discontinuities observed when spherical gold particles are manipulated on rigid substrates by AFM.

#### B. Influence of the shape

**Figure 4:**
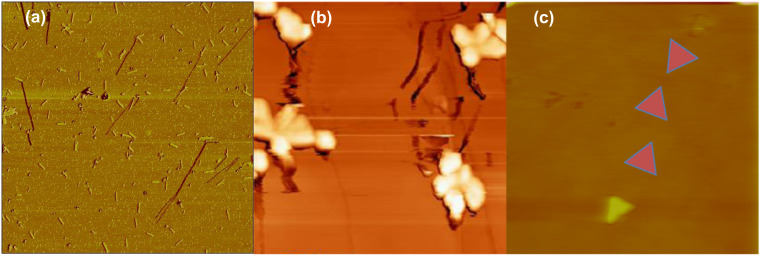
AFM images of nanocluster movement during their manipulation (a) gold nanorods deposited onto silicon wafer, scan size: 12 µm; (b) antimony islands on HOPG, scan size: 1.5 µm; (c) Au nanotriangles on silicon wafer. Middle triangles have been intentionally colored in to illustrate the trajectory of the Au nanoparticles during manipulation, scan size: 5 µm.

The manipulation of spherical and asymmetrical nanoparticles by AFM represents a way to understand and control the motion of complex shaped nanoparticles. For instance, manipulation of elongated objects such as rigid Au nanorods induces mainly sliding and rolling of the nano-objects, and this movement varies with the different stages of nanomanipulation time scale. As shown in Figure 4, the rods first tend to move perpendicular to their principal direction of motion and then wobble along their longitudinal axis. The average orientation of the rod is perpendicular to its direction of motion. According to theoretical simulation and experience, the torque applied by the tip to the rods results in a wobbling motion, which has no determining influence on the overall direction of the nanoparticles [39].

For triangular and flower shaped nanoparticles, the nano-objects mostly evolve through a translation movement, as well as a rotation along their main perpendicular axis during the manipulation, as shown in Figure 4. While asymmetric particles wobble around a fixed angle, they do follow a well defined path with a specific angle. Simulation of the trajectory of these different particles is still under progress [40] and may lead to a better understanding of how to induce a well-defined direction of motion to nanoparticles by adjusting the operating parameters of the AFM. Besides the shape and the size of the particles, the chemistry of the functional grafting surrounding the particle also strongly affects their movement and trajectory during nanomanipulation.

### 2. Influence of the chemistry of the particles on a flat substrate

**Figure 5:**
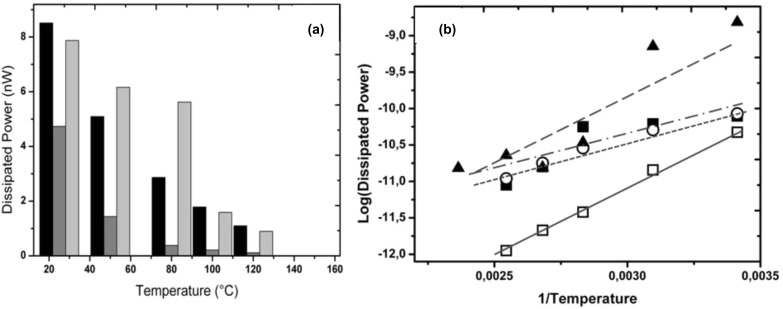
(a) Average power dissipation accompanying the onset of motion of as-synthesized and coated nanoparticles on silicon in air vs temperature. *Black columns*: as-synthesized NPs that are uniformly distributed on the substrate, *dark gray columns*: CH_3_-coated NPs, *light gray columns*: as-synthesized NPs randomly distributed on the substrate. (b) Logarithm of the dissipated power in moving as-synthesized and coated NPs on silicon wafer vs reciprocal temperature. *Closed squares*: as-synthesized nanoparticules, *open circles*: as-synthesized nanoparticles ordered organized, *open squares*: CH_3_-coated nanoaprticles, *closed triangles*: OH-coated nanoparticles.

Because real surfaces are often heterogeneous in their chemical composition, functionalized nanoparticles provide good model systems to study and tune the mobility of nano-objects on these substrates. As a next step, the role of the hydrohilicity and hydrophobicity of the functional grafting on spherical Au nanoparticles is illustrated in Figure 5a. This series of experiments was performed on a Veeco AFM whose tip follows a zigzag scan path.

The role of the hydrophobic or hydrophilic character of the interface in the manipulation process was investigated, using gold nanoparticles bearing OH- and CH_3_-terminated thiol groups (as described in the Experimental section) and moving these particles against a flat bare silicon substrate. The results are summarized in Figure 5 which displays the average power dissipation required to induce the motion of the particles. The first observation that arises directly from this figure is that the presence of a hydrophobic interface significantly enhances the mobility of the particles. The energy required to move OH-coated nano gold particles was found to be at least 10 times higher than that for CH_3_-coated particles. We also observed that the manipulation of hydrophilic coated nanoparticles often results in a damage to the tip due to the high particle–substrate adhesion force. This strong adhesion between silicon substrate and hydrophilic coated nanoparticles primarily arises from intermolecular interactions. It may also involve a contribution from capillary bridges between the substrate and the NPs on one hand and between the closest NPs on the other hand (see below, subsection 5). In contrast, it has already been observed that the thin adsorbed water film formed on the silicon wafer acts as a lubricant when confined between the hydrophobized CH_3_-coated nanoparticles and the (hydrophilic) substrate [41–43].

As we can see here, the eventual role of relative humidity (*RH*%) which is an environmental parameter, strongly depends on the chemistry of the NP–substrate interface. Another environmental parameter, namely temperature, also affects the mobility of the nanoparticles. The influence of extrinsic (environmental) parameters is discussed in the following paragraph.

### 3. Influence of the temperature

Figure 5a shows a histogram of the raw values of power dissipation vs the temperature for temperatures ranging from 20 to 150 °C. These results clearly show that the power dissipation involved in the motion decreases with the temperature. This effect appears to be stronger on hydrophilic particles. Intuitively, one could expect this result since the higher thermal energy (*k*_B_T) impedes the formation of stable intermolecular bonds and water bridges between particles and substrate, reducing the adhesion between them. Similar thermal effects have been recognized in friction on hydrophilic surfaces measured with different scan velocities [42]. It is worth noting that during this temperature dependent manipulation no evident damage was observed on working areas.

Figure 5b shows a logarithmic plot of the dissipated power as a function of the reciprocal temperature. The experimental data of all NP–substrate couples can be fitted well using a linear regression (r² > 0.90), except the data of as-synthesized NPs for which r² is ~0.78. This linear behavior of [log(dissipated power)] vs (1/T) actually corresponds to an exponential decay of the dissipated power with T which points to a thermally activated process [44]. The slopes of these linear fits correspond to (Δ*E*_act_/*k*_B_), where Δ*E*_act_ represents an activation energy barrier with respect to a reference state *E*_0_: Δ*E*_act_ = (*E*_0_−*E*_act_) where *E*_act_(*T*) is the energy input involved in the motion of the particle. This energy variation (slope) is high for the CH_3_-hydrophobized NPs, indicating a strong decrease of the input energy with the temperature which would be expected for low adhesion strength between nanoparticle and substrate. Surprisingly, a quite similar behavior in, both, trend and activation barrier of the temperature-dependent mobility is observed for the hydrophilic OH-coated NPs. An explanation for this result may come at least partly from the complex behavior of the adsorbed (structural) water depending on temperature in the hydrophilic system. Beyond the observed – and rather reasonable – general trend, the strong decrease with the temperature of the energy required for particle movement, the magnitude of the activation barrier for essentially hydrophilic and hydrophobic contacts will certainly need further confirmation experiments, as well as a more extensive interpretation. Indeed, we assumed in our treatment (Figure 5b) ideal Arrhenius behavior where the activation energy is independent of the temperature in both systems. This is an assumption which may not be the case for the complex water bridging hydrophilic contact.

### 4. Organization effects

**Figure 6:**
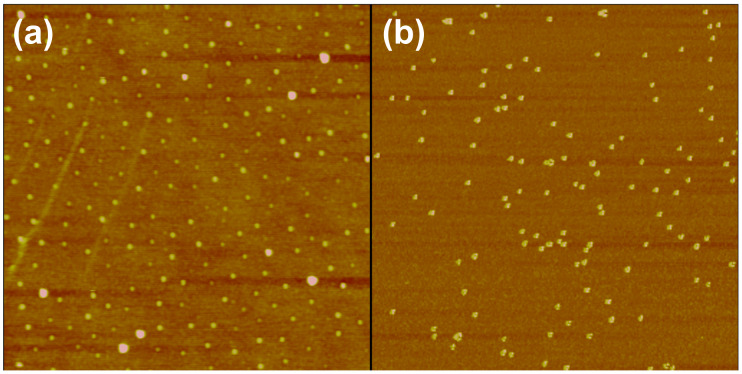
AFM images of 25 nm diameter gold nanoparticles deposited onto a silicon wafer. (a) Ordered organization as described in the Experimental section, (b) random distribution. Frame sizes: 3 µm and 1 µm, respectively.

The first and third columns of the series shown in Figure 5a show the threshold power dissipation for the motion of randomly and ordered organized distribution of nanoparticles (see Figure 6), obtained as described in the Experimental section.

The power dissipation at different temperatures is comparable in both cases. This result can be explained by the average distance between the nano-objects, which is 70 nm for the random distribution and 100 nm for the ordered one. At such a scale, the interparticular forces are of the order of long range interactions. The mobility of particles is essentially affected by electrostatic interactions arising from residues from the synthesis (citric acid) that may be adsorbed on the particles. It is thus normal, in the absence of both physical contact and notable intermolecular forces between the particles, that their mobility is independent of their organization (random or ordered). In other words, this result means that as long as the particle number density *n*_p_ is such that the interparticle distance *d*_p_ ~ (*n*_p_)^−1/2^ is larger than the range of short-ranged forces [45], their mobility is not affected by their mutual intermolecular binding and is thus independent of their organization. It is worth noting that this absence of true intermolecular binding does not exclude possible particle–particle interaction through capillary forces arising from nanosized condensation films connecting particles at these separations.

### 5. Influence of humidity and vacuum environment

#### A. Effect of relative humidity

**Scheme 2:**
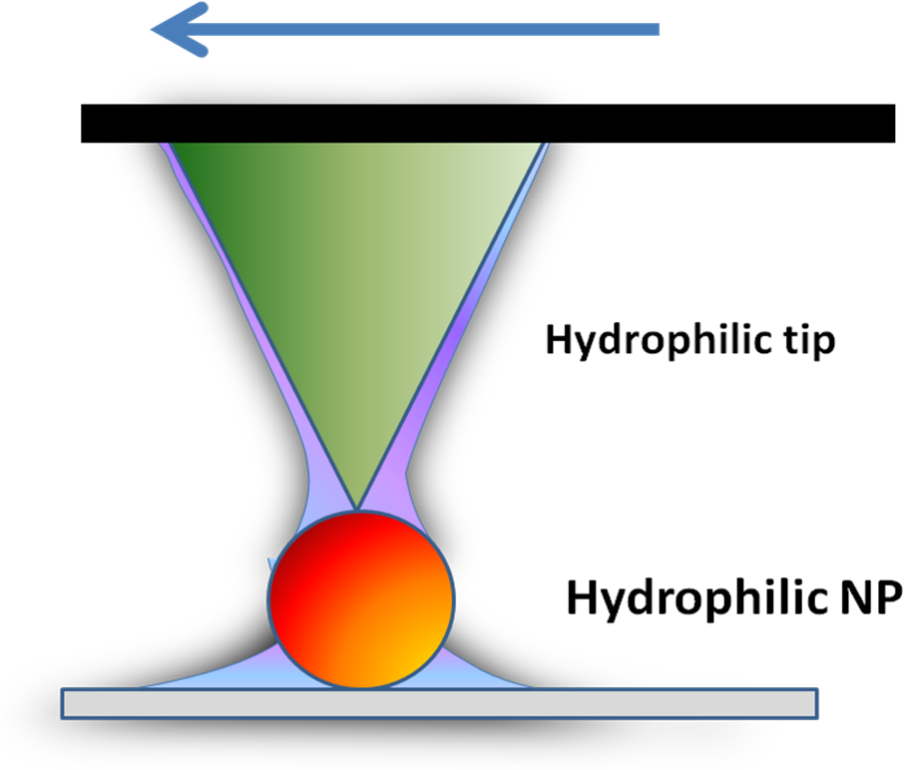
Formation of two capillary water bridges between hydrophilic tip and particle, and particle and surface.

The presence of surface contaminants (dust or water) affects the mobility of nanoparticles as this directly changes the intermolecular interactions between the nanoparticles and the surface. As it has been discussed in subsection 2, a contribution from capillary bridges has also a strong influence on the mobility of spherical Au nanoparticles during their manipulation. Indeed, capillary forces of water films between both interfaces, nanoparticle–surface and tip–nanoparticle, will depend on the volume of liquid condensate present at the interface, as well as the interface geometry [46] (see Scheme 2). The presence of the water meniscus at both interfaces will increase the adhesive forces and lower the mobility of the NPs.

In this section we describe our studies on the mobility of as-synthesized Au spherical NPs and CH_3_-coated ones. The diameter of the Au nanoparticle is about 20 nm. The ambient (*RH* = 33%) and higher relative humidity results displayed in Table 1 illustrate how the adsorption of water on nanoparticles can affect the adhesion and friction forces at, both, tip–nanoparticle and nanoparticle–surface contacts. Independently of the environmental conditions, manipulation of nanoparticles on a surface requires that they are loosely attached in order to be able to move them.

**Table 1:** Mobilities of spherical Au nanoparticles (hydrophilic and hydrophobic) versus humidity rate during their manipulation using an AFM in tapping mode (zigzag scan path).

Relative humidity (%)	33 (ambient conditions)	43	53

**as-synthesized Au NPs**	movement	fixed	fixed
**CH_3_-coated Au NPs**	movement	movement	movement

The decrease of relative humidity from 53 down to 33% has a strong impact on the mobility of the hydrophilic Au NPs. Above *RH* = 43%, the adsorbed Au particles do not move, because the energy transferred from the tip to the particle during the tap is not high enough to break the capillary bridges formed at both interfaces. As a consequence, the overall energy does not reach the threshold barrier to move the particle and is completely dissipated in the system.

**Scheme 3:**
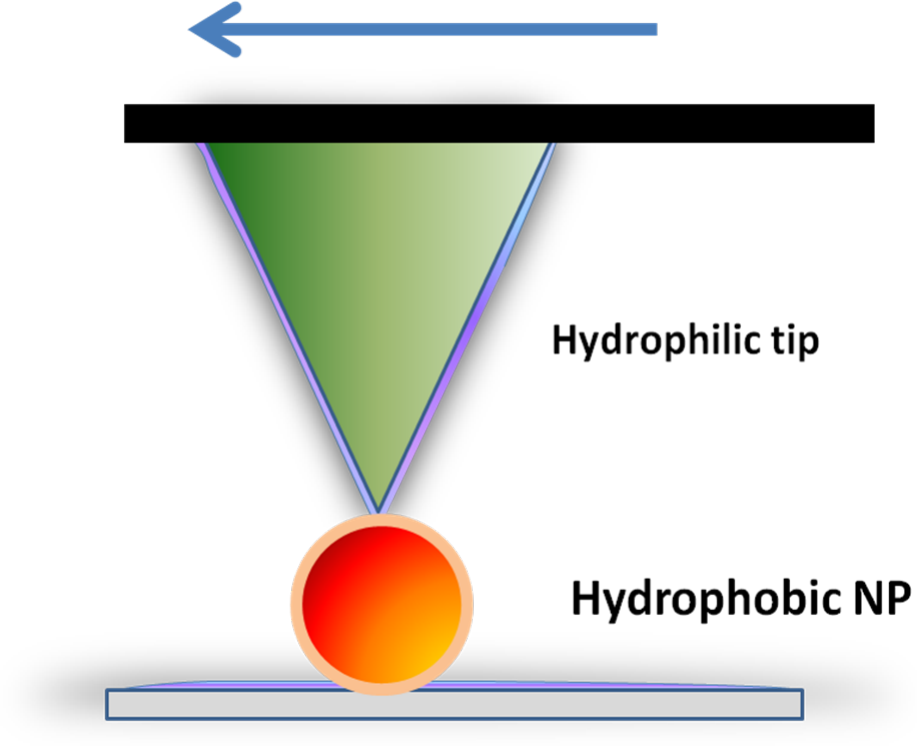
Formation of two water layer films between hydrophilic tip–hydrophobic particle, and hydrophobic particle–hydrophilic surface, respectively.

However, this process does not affect strongly the mobility of hydrophobic Au NPs. They move whatever the environment. This difference can be explained by the existence and the local shape of a liquid condensate (Scheme 2 and Scheme 3) around the tip–substrate contact [47].

In a humid environment, the magnitude of friction and adhesion forces is strongly dependent on the capillary force that is related to the intrinsic wetting properties of the interfacial system. As a consequence, the resulting water meniscus (or layer) can either increase friction through increased adhesion in the contact zone (hydrophilic interfaces) or reduce it through the lubricating effect of a water layer.

Further experiments should also prove that the bigger the particles are, the higher the capillary effect will be as has previously been observed in contact mode [35–48].

#### B. Vacuum environment

**Figure 7:**
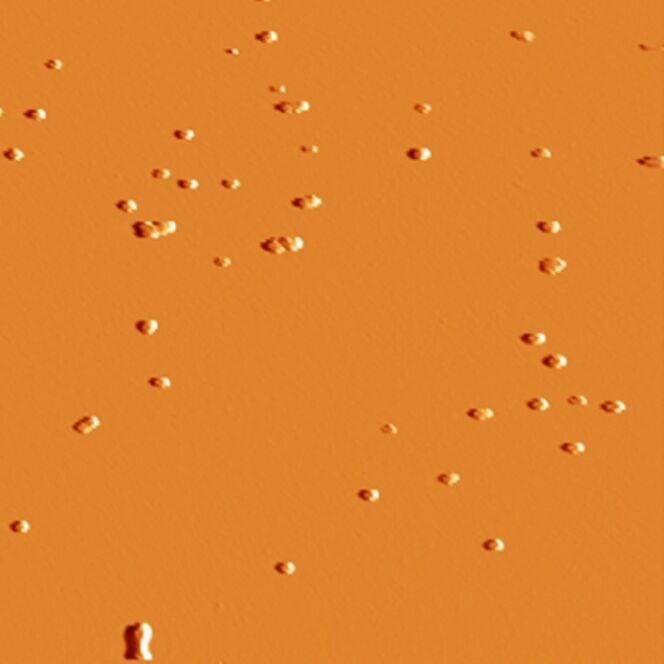
As-synthesized Au particles on silicon in ultra-high vacuum. Frame size: 3 µm.

The environment is a crucial parameter in manipulation (tribological) experiments. The adhesive and frictional results are directly dependent on the humidity and temperature of the surrounding medium. Concerning the influence of humidity (or more exactly the absence of humidity), we have investigated how the nanomanipulation process is affected in ultra high vacuum (UHV) environment. The topography image in Figure 7 shows the gold particles on a silicon substrate after the sample was transferred into UHV without any further treatment, which could have changed the organization of the particles. The shape of the particles is well defined, and the structure of some aggregates can be recognized, due to the absence of convolution effects that usually arise from the water layer which may cover the particles under ambient conditions. This image thus shows that the transfer into UHV by itself does not affect the shape of the NPs or their organization. When manipulated under UHV conditions, the particles could not be moved, even when imaged at the maximum magnification available with our system (in the order of 100 nm). Even in contact mode, with forces of a few nanonewtons applied to the particles, no motion was observed. This UHV result particularly illustrates the important lubricating role of the adsorbed water layer between the particle and the substrate in both the free (Brownian) and externally-driven motion of nanoparticles.

The previous sections have demonstrated the influence of the morphological, environmental and chemical parameters on the mobility and movement of the particle. The following addresses the influence of the topography of the substrate.

### 6. Influence of the topography of the substrate

Manipulation of gold nanoparticles was investigated on flat bare silicon wafers, as well as on nanostructured (or nanopatterned) silicon wafers, i.e., silicon substrates that are patterned on the nanoscale.

**Figure 8:**
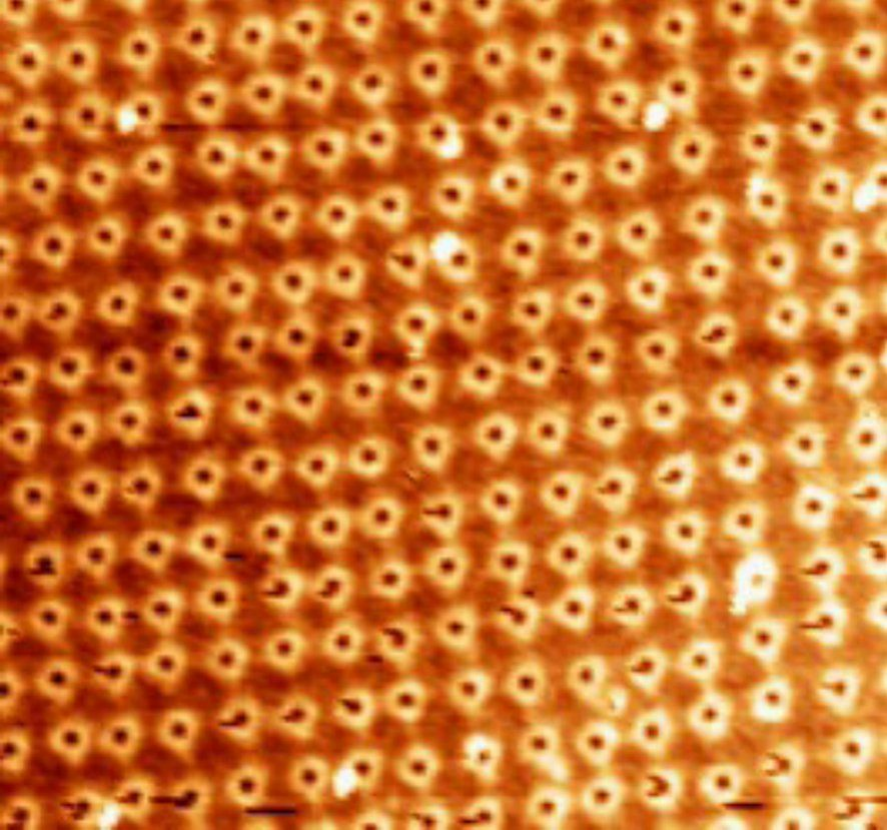
AFM image of nanopatterned surface exhibiting Si pits: Frame size: 3 µm.

The following experiences were performed using a raster scan path of the tip mounted on a Nanosurf AFM. On flat bare silicon wafer, the direction of motion of the 25 nm diameter gold nanoparticles was initially well defined, but changed after acquiring a couple of images. This makes it much more difficult to move the particles, even for higher values of the drive amplitude, possibly because of tip contamination. Hence, the idea to modify the topography of the surface was chosen to study the effect of the geometrical surface confinement on the mobility and trajectory of the nanoparticles. Nanopatterned substrates shown in Figure 8 were chosen for that purpose.

**Figure 9:**
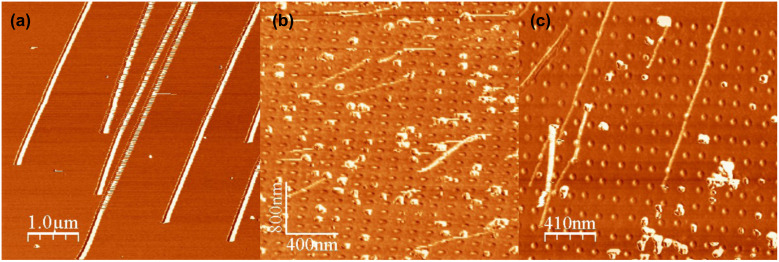
Manipulation of as-synthesized Au nanoparticles on (a) a flat silicon wafer with a spacing of 9.7 nm and (b) a nanopatterned one with a spacing 16 nm, and (c) a patterned wafer with a spacing of 3.9 nm.

The surface patterns consist of an array of nanopits created by the focused ion beam (FIB) milling technique. The width and depth of the pits are 650 nm and 5 nm, respectively, and the spacing between two adjacent pits is 125 nm. On the patterned surface, the mean direction of motion remains identical (on average), even after a long acquisition time. This stability of the direction of the particle movement observed here on the nanopatterned substrates can be attributed to "self-cleaning" of the tip when it crosses the shallow pits. Considering that the pits have only a small influence on the particle direction (Figure 9), which means that all the particles follow the same direction, this parameter could be ignored for determining the deflection angle. As a result, patterned surfaces were chosen for this determination, rather than the flat bare silicon surfaces. The influence of the spacing *b* separating two scan paths on the deflection angle has been shown by simulation of these experiments [39–49]. Figure 9b and Figure 9c display the change in angle for the same surface and identical particles for b = 16 nm and 3.9 nm, respectively. The trend of adopting higher angles with lower spacing is clear from these results. To confirm the topographical effect, as-synthesized Au NPs were also manipulated on different substrates such as nanopatterned silicon wafers presenting grooves, and steeped HOPG surfaces [40].

Manipulation experiments were repeated to check the influence of the deep grooves (either on Si wafers or on HOPG) on the trajectory of the moving particles. It was found that the deep grooves slightly influence the direction of movement of the particles as particles tend to follow their preferential angle during movement.

Finally, the last important and technological parameter of AFM nanomanipulation is the effect of scan velocity on the movement of the nanoparticles.

### 7. Influence of scan velocity

The inﬂuence of the sliding velocity on friction, which accounts, at least partly, for the dynamical response of the boundary layer, can be exploited to gain insight into the manipulation of nano-objects [41].

Spherical particles (as-synthesized Au NPs) of 35 nm and 60 nm in diameter were moved in tapping mode with Veeco AFM following the previous procedure described in subsection 1. The drive amplitude threshold to move the particle was recorded as well as the phase shift to estimate the loss of energy during the movement of the particles. These experiments were repeated for different scan tip velocities ranging from 0.1 up to 10 µm·s^−1^ on three model substrates, i.e., a cleaned silicon wafer (SiO_2_), and two other ones, coated with either hydrophilic (–NH_2_) or hydrophobic (–CH_3_) self-assembled monolayers.

**Figure 10:**
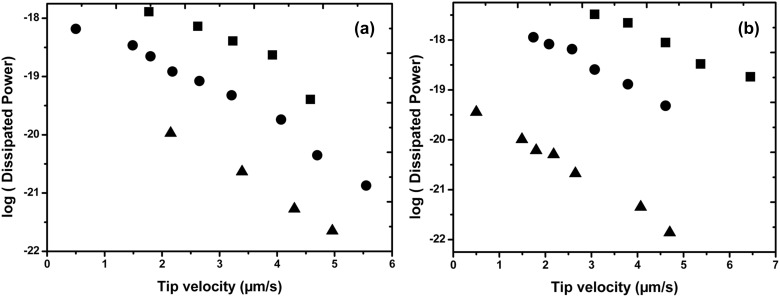
Logarithm of the dissipated power in moving as-synthesized NPs on silicon wafer versus the tip scan speed. Substrates: *circles*: SiO_2_ silicon wafer; *squares*: NH_2_-coated silicon wafer (hydrophilic substrate); *triangles*: CH_3_-coated silicon wafer (hydrophobic substrate). (a) 35 nm diameter Au NPs, (b) 60 nm diameter Au NPs.

The results of the velocity-dependence of the dissipated power are plotted in Figure 10. The dissipated power has been plotted on a logarithmic scale to allow a more usual comparison with the literature [14,21,42]. To ensure that the measured power dissipation was representative of the spherical gold nanoparticles motion, several particles (at least 10) were moved under similar conditions.

Our results in Figure 10 show that for both nanoparticle sizes (35 and 60 nm), the dissipated power during the tip–particle contact depends on the chemical nature of the substrate. The magnitude of the dissipated energy gradually and significantly increases from the more hydrophobic to the more hydrophilic substrate as one could expect from the intermolecular interactions involved at the different interfaces. This dissipated power also increases with the diameter of the nanoparticles as expected from the increase of the NPs–substrate contact area.

At the more hydrophobic substrate (CH_3_), the interactions with the hydrophilic nanoparticles (as-synthesized citrate stabilized NPs) mainly involve London dispersion forces that have a much lower magnitude as compared to the polar, hydrogen and electrostatic bonds involved in the adhesion of these citrate-stabilized nanoparticles, with more hydrophilic (SiO_2_ and NH_2_) substrates. The maximum dissipated power appears for the more polar substrates. It is worth noting that this value can involve a contribution from the capillary water bridges which readily form on more hydrophilic systems under ambient conditions as previously discussed in subsection 5A. It is also worth noting that we also verified here that both the surface and the particle were free of any observable damage after each manipulation.

However, independent of the nature of the intermolecular interactions exchanged between tip and nanoparticles or nanoparticles and surface, and independent of the size of the spherical particles, the logarithm of the dissipated power during the manipulation systematically decreases linearly, when the scan velocity increases. This linear dependence is generally attributed to a decrease of the energy dissipation in the contact as the velocity increases, in a way similar to the velocity (frequency)-dependent viscoelastic and/or plastic dissipation in polymers (as well as metals), as is described for instance through the time-temperature superposition principle for polymers [50–52]. However, from this discussion, it appears that further investigations regarding the velocity dependence of the dissipated power are still necessary on both experimental and theoretical levels. This work is now under investigation and we hope to be able to give an additional and detailed explanation regarding the mechanisms from our experimental results.

## Conclusion

The manipulation of nano-objects is still a relatively rare operation. Because micro/nanomechanics has not been completely well-developed, two-dimensional positioning of nanometer-size particles on a substrate at ambient conditions remains a difficult operation and depends on several critical physical, mechanical and chemical parameters. However, advances have enabled better control in nanoscale manipulation. In this paper, we have described manipulation of gold colloidal nanoparticles using AFM in tapping mode. The inﬂuence of structural characteristics of the particle (chemistry, size, shape) and the substrate (chemistry and topography) have been investigated. It has been shown that the mobility of the particles was significantly affected by the nature of intermolecular tip–particle and particle–surface interactions, the particle shape and size, the operating environment conditions (relative humidity *RH*% and temperature *T*), as well as the tip scan velocity. The dissipated power during manipulation was quantified under various operating conditions (*RH*%, *T*, tip scan speed). Our experiments show that the velocity dependence of the dissipated power at these nanoscale contacts is far more complex than what one could predict, based on the sole contribution of the tap energy and capillary liquid bridging adhesive force. Indeed, the thermal energy produced within the tip–substrate contact can induce molecular excitations and structural transitions in the topmost contacting layers, the magnitude of which also increases with the sliding velocity. Direct access to the nanoscale contact between tip and nanoparticle, and nanoparticle and surface are limited with the current device, thus any quantitative analysis of these results remain at this stage scientifically debatable. The second difficulty is naturally related to the yet insufficiently understood size effects that show up in nanoscale friction and strongly affect the results. In addition, real-time monitoring of the manipulation process is almost impossible. Most of the time, imaging is *offline* and the unexpected problems during pushing cannot be detected. Another way is utilizing the force feedback information during pushing for reliable manipulation. This is currently being seriously investigated and correlated to theoretical studies [20]. Because of potential improvements in the mechanical and theoretical fields, more complex and precise manipulations of particles, molecules and single atoms at surfaces using AFM will become achievable and nanoscale manipulations may be of fundamental importance for the realization of nanoscale devices in the future.

## Experimental

Gold nanoparticles were adsorbed onto silicon wafers and manipulated in AFM tapping mode. They were either bare or coated with self-assembled monolayers terminated with hydrophobic (methyl, –CH_3_) or hydrophilic groups (hydroxyl, –OH).

### Bare gold nanoparticles

**Figure 11:**
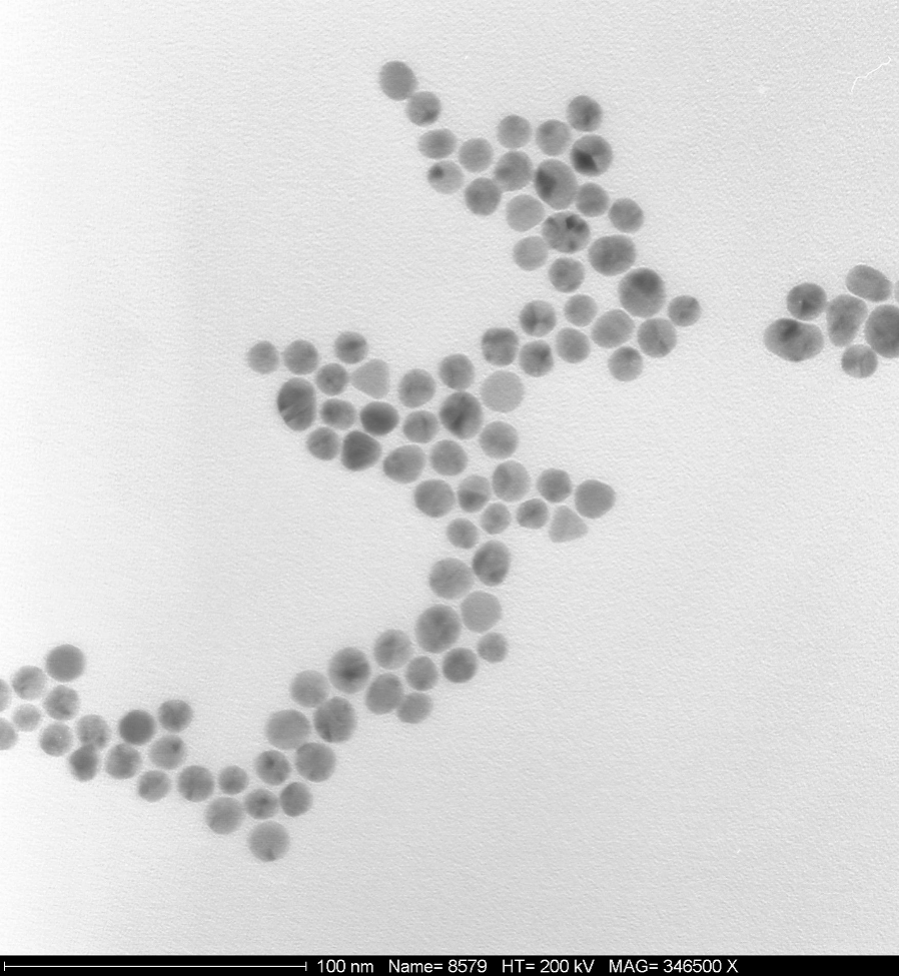
400 nm × 400 nm TEM image of 25 nm diameter gold nanoparticles.

The colloidal suspension was made by reduction of an aqueous solution of nanogold particles, HAuCl_4_·3H_2_O supplied by ABCR, Karlsruhe, Germany. The suspension was stabilized with citric acid trisodium salt (Aldrich), which, by reducing HAuCl_4_, imparts the negative charge of the citrate ions to the gold nano-particle surface [27–28]. The average size of these nanogold particles, as determined from transmission electron microscopy (TEM) images, was 25 ± 5 nm (Figure 11).

### Coated gold nanoparticles

Dodecanethiol for methyl terminated monolayers and 11-mercapto-1-undecanol for hydroxyl terminated monolayers were obtained from Sigma-Aldrich and used as received. Hydroxyl or methyl-thiol-stabilized gold nanoparticles were synthesized according to a modified version of two common syntheses [21]. The as-synthesized nanosphere solution [27–28] was centrifuged at 7000 rpm for 20 min to pellet the nanoparticles, decanted, and then re-suspended in 1 mL of deionized water to reduce the citric acid concentration. The nanoparticles were then purified from excess surfactant and other reactants by dialysis for one week. Finally, the dialyzed solution was centrifuged and particles were re-dispersed in tetrahydrofuran. 300 µL of the appropriate thiol (methyl- or hydroxyl-terminated) was added to the solution, sonicated and stirred for approximately 2 h to allow the grafting reaction to reach completion. The yellow colored solution slowly became colorless was stored at 4 °C until required. The average diameter of the synthesized nanoparticles is 25 ± 5 nm.

### Nanoparticles adsorption

#### Random adsorption

For the adsorption experiments, a concentration of 0.03 wt % of nanoparticles in the aqueous or organic dispersion was used. The experimental protocol basically involved the particle adsorption by immersing the samples for about 20 minutes in the suspension, whose temperature was maintained at 20 ± 1 °C. After this initial adsorption stage, the samples were removed from the bath, and the thick dispersion film remaining at the substrates was allowed to dry.

#### Ordered organisation

Samples were provided by McFarland’s group at UCSB. Au nanoparticles (25 nm diameter) were synthesized as described previously [27–28]. The Au NPs coated silicon wafer was prepared using a micelle encapsulation method [53–54]. Au nanoparticles were encapsulated by diblock copolymer poly(styrene)-*block*-poly(2-vinylpyridine). The solution was deposited onto silicon wafer and dried under a nitrogen flow. After being dip-coated, the polymer was removed by oxygen plasma treatment (see Figure 6).

#### Self-assembled monolayer coated silicon wafer

The molecular surfaces were prepared by self-assembling organosilane molecules onto silicon wafers Si(111) with a native thin oxide (SiO_2_) layer of ~1.5 nm. The organosilane compounds were methyl terminated hexadecyltrichlorosilane (–CH_3_), and the amine terminated 6-aminohexylaminopropyltrimethoxysilane (–NH_2_). Homogeneous films were obtained by vapor-phase deposition in a dynamically evacuated chamber (1 h at 10^−3^ torr), using a mineral oil as dispersing solvent for the molecules. This consists of mixing the organosilanes in paraffin oil before evacuating the atmosphere in the dessicator enabling the molecules to pass into the vapor phase and stick to the substrate placed above the mixture [33–50].

### Manipulation Setup

#### In-air measurements

The images in air were acquired with two commercial AFMs (Multimode, Nanoscope IV from Veeco and Mobile S from Nanosurf). Rectangular silicon cantilevers with resonance frequencies *f*_0_ around 120 kHz and 190 kHz, quality factors of around 800 and 600, and nominal spring constants of 5 and 48 N/m (respectively, MPP12100 from Veeco and PPP-NCLR from Nanosensors) were used. During manipulation, the oscillation amplitude of the tip, *A*_set_, was kept constant by a feedback loop. In this case, the power dissipation accompanying the tip–sample interaction can be determined from Equation 1 [36].

#### UHV measurements

The images in UHV were acquired with a custom built AFM available at the University of Basel [21]. The base pressure was below 10^−9^ mbar. Due to the high quality factor in UHV, the out-of-contact-resonance frequency shift was used as the imaging parameter instead of the tip's oscillation amplitude (NC-AFM). We have also performed measurements in contact mode, where the set point is determined by the normal load acting between tip and sample. PPP-NCLR and CONT cantilevers from Nanosensors were used in both cases.
